# Mitochondrial function and intracellular distribution is severely affected in in vitro cultured mouse embryos

**DOI:** 10.1038/s41598-022-20374-6

**Published:** 2022-09-27

**Authors:** Marta Czernik, Dawid Winiarczyk, Silvestre Sampino, Paweł Gręda, Salvatore Parillo, Jacek Andrzej Modliński, Pasqualino Loi

**Affiliations:** 1 Institute of Genetics and Animal Biotechnology, Polish Academy of Sciences, Jastrzębiec, ul. Postepu 36A, Poland; 2 Faculty of Veterinary Medicine, University of Teramo, Via Balzarini 1, Teramo, Italy; 3 Department of Morphological Sciences, Faculty of Veterinary Medicine, Warsaw University of Life Sciences, Warsaw, Poland

**Keywords:** Embryogenesis, Mitochondria

## Abstract

Studies of mitochondrial dynamics have identified an intriguing link between energy supply balance and mitochondrial architecture. This suggests that inappropriate culture conditions might inhibit mitochondrial functions, and affect embryonic development. Therefore, this study was conducted to determine whether in vitro culture (IVC) might affect mitochondrial function, distribution, organization (by Mitotracker Green), gene expression on RNA level (by qPCR), and protein expression and localization (by western blot and immunostaining) involved in regulation of mitochondrial functions. Mitochondria in 2-cell IVC embryos were less numerous compare to IN VIVO while the localization and distribution do not differ between the groups. Mitochondria of in vivo blastocysts formed elongated network along the cells, while in IVC were fragmented, rounded, and aggregated mainly in the perinuclear region. Additionally, mitochondria of IN VIVO embryos moved back and forth along their long axis on radial tracks, while in IVC blastocysts were much less active. mtDNA copy number in IVC blastocysts (92,336.65 ± 5860.04) was significantly lower than that of IN VIVO (169,103.92 ± 16,322.41; P < 0.02) as well as lower protein expressions responsible for mitochondrial fusion was observed in IVC blastocysts. Results indicate that in vitro culture affect on perturbations in mitochondrial number and function, which is associated with decreased developmental competence of in vitro produced mouse embryos.

## Introduction

Mitochondria are organelles, whose functional integrity is essential for cellular survival. They are surrounded by two membranes, contain their own DNA (mtDNA), and act as the energy producers of the cell via oxidative phosphorylation.

There are a number of reports showing that mitochondria and mtDNA have a crucial impact on early embryonic development^1–3^ It has been shown that inappropriate mitochondrial activity at the pronuclear stage is associated with early developmental arrest^4^, likely linked to decreased expression of mitochondrial genes^5^. In addition, the mitochondria play a vital role in the oocyte cytoplasm, for they provide adenosine triphosphate (ATP) for fertilization and later for preimplantation embryonic development. They also act as stores of intracellular calcium and pro-apoptotic factors. Recent studies have shown that mitochondrial dysfunction, such as structural, spatial, and genetic abnormalities in early embryos^6^, might influence normal embryonic development as well as fetal and placental development^7,8^. Of relevance to our work, it has been shown that inappropriate in vitro culture conditions (as components of culture medium, oxygen concentation) might affect the development and function of mitochondria^9^.

An interesting feature of the early embryo is that between fertilization and implantation it depends on the function of existing mitochondria present at ovulation^10^. As embryo cleavage progresses, the total number of mitochondria within each blastomere decreases. At this stage, the blastomeres do not express mitochondrial replication factors^11^. Consequently, late preimplantation embryos contain smaller number of mitochondria per cell compared to the zygote. As a result, any adverse influence on mitochondrial function (e.g., increased mutational load in the mtDNA) will negatively impact the development of the pre- and post-implantation embryo^12^.

Moreover, mitochondria exhibit a unique quality maintenance function. They have numerous stages of fusion and fission. If active, mitochondria maintain a polarised membrane, and fuse with other mitochondria to transfer components and maintain or improve the function of damaged or poorly performing organells^13^. However, if the mitochondria are non-functional and their membranes are depolarised, they do not fuse with active mitochondria and are targeted for removal via mitophagy. This process prevents the mixing of damaged and high-quality mitochondria and decreases the pool of poorly performing mitochondria^13^.

In mammals, three proteins are required for the fusion process. Two mitofusins: mitofusin 1 (Mfn1) and mitofusin 2 (Mfn2), are responsible for fusion of the outer membrane, while a single dynamic family member, Opa1, is required for inner membrane fusion. It is known that Mfn2, which plays such a central role in the fusion process, is essential for embryonic development^7^. Additionally, low expression of Mfn2 attenuates blastocyst formation rate and cleavage speed in mouse zygotes and causes mitochondrial dysfunction, as confirmed by ATP and mtDNA levels, and mitochondrial membrane potential^14^.

The present study was conducted to investigate the developmental effects of in vitro culture on mitochondrial distribution, functionality, motility, membrane potential, copy number of mtDNA, gene expression of mitochondrial fusion-related proteins and their localization.

## Results

### Effects of in vitro culture on developmental competence of mouse embryos

**Figure 1:**
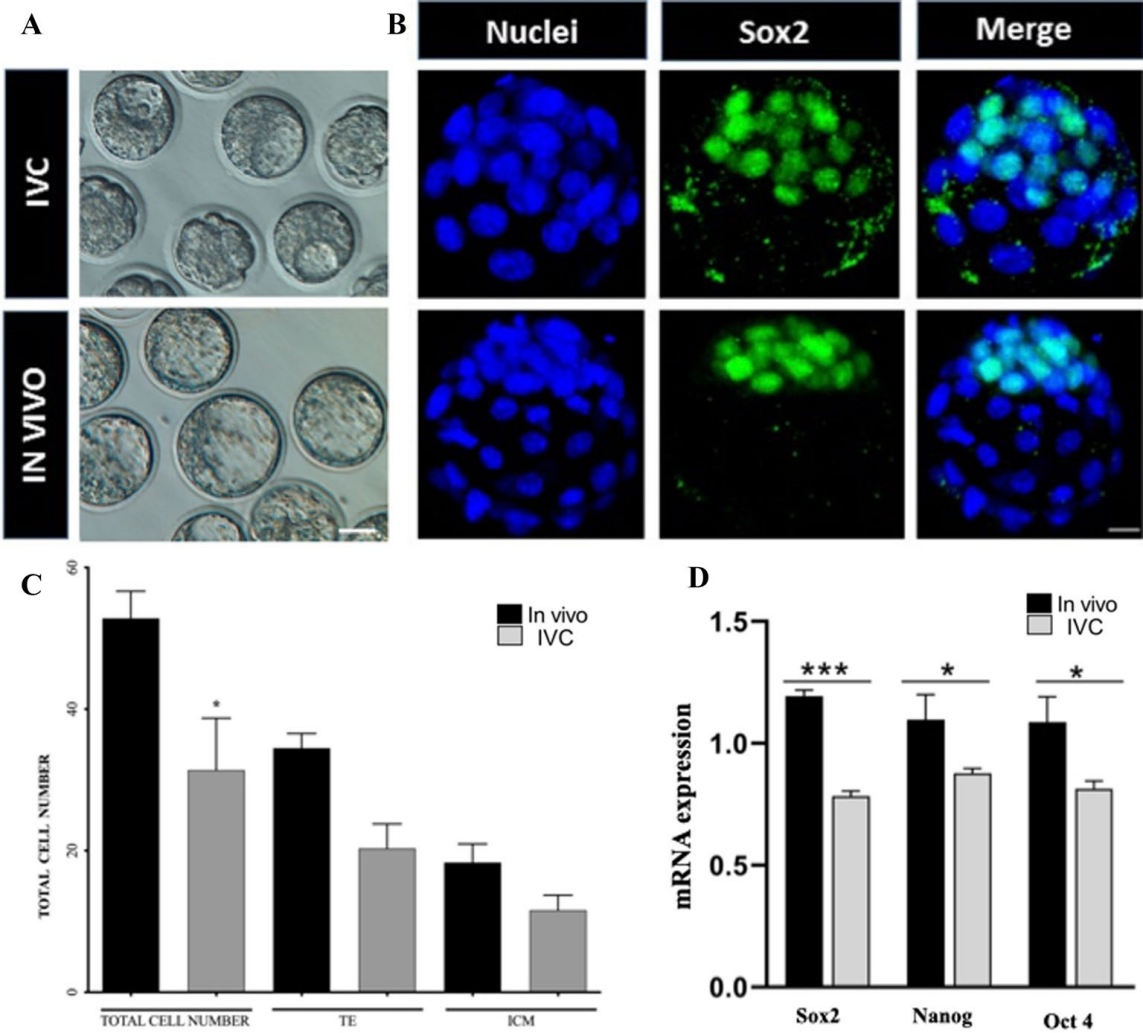
Representative image of IVC (upper line) and IN VIVO developed (bottom line) blastocysts at stage (E.3.5) in bright field (left) (**A**) and after staining with Sox2 [green; inner cell mass (ICM)] and Hoechst 33,342 (total cell number, nuclei) (**B**); (**C**) Graph represent total, ICM and trophoblast cell number in IVC and IN VIVO developed embryos at the blastocyst stage (E.3.5). Scale bars: 30 µm (bight field blastocyst) and 20 µm (immunoassayed blastocyst); *mean p < 0,05; (**D**) Sox2, Oct-4 and Nanog gene expression detected by qPCR in blastocysts stage embryos; Sox2 ***p < 0.0001, Nanog *p = 0.0217, Oct4 *p = 0.0122.

The purpose of the assessment was to evaluate whether in vitro culture (IVC) affects embryo development. To do that, mouse zygotes were cultured in standard IVC medium (KSOM) till E3.5 (to the early blastocyst stage). Same-day IN VIVO developed embryos were flushed, and blastocyst rate was evaluated in both groups. Results showed that IVC blastocyst formation rate (78%, 106/136) was lower than when developed IN VIVO (98%, 100/102; P < 0.05). Moreover, microscopical observation shown that quality of the IVC blastocysts was much lower than that of the IN VIVO control group (Fig. 1A). Moreover, the blastocysts were stained with Sox2 and Hoechst to distinguish the inner cell mass (ICM) and total cell number (Fig. 1B). Results shown differences between the groups (Fig. 1C). The total cell number (31 ± 1.1 vs. 52 ± 2.3), inner cell mass (18.3 ± 0.75 vs 11.6 ± 0.67) and trophoblast (34.5 ± 1.1 vs 20.3 ± 0.6) cells number in IVC blastocysts were significantly lower than the IN VIVO developed blastocysts (Fig. 1C; P < 0.05). For deeper analysis of the embryo quality we assessed the mRNA expression level of pluripotency markers, including Sox2, Oct-4 and Nanog. All mRNA levels was statistically lower in IVC blastocysts when compare to the IN VIVO group. (Fig. 1D). These results confirm that in vitro culture negatively affects embryos development in terms of cell number, their distribution and pluripotency genes expression.

### In vitro culture effects on mitochondria distribution and their activity in mouse embryos

We next evaluated the effects of IVC on mitochondrial distribution using MitoTracker Green.

**Figure 2:**
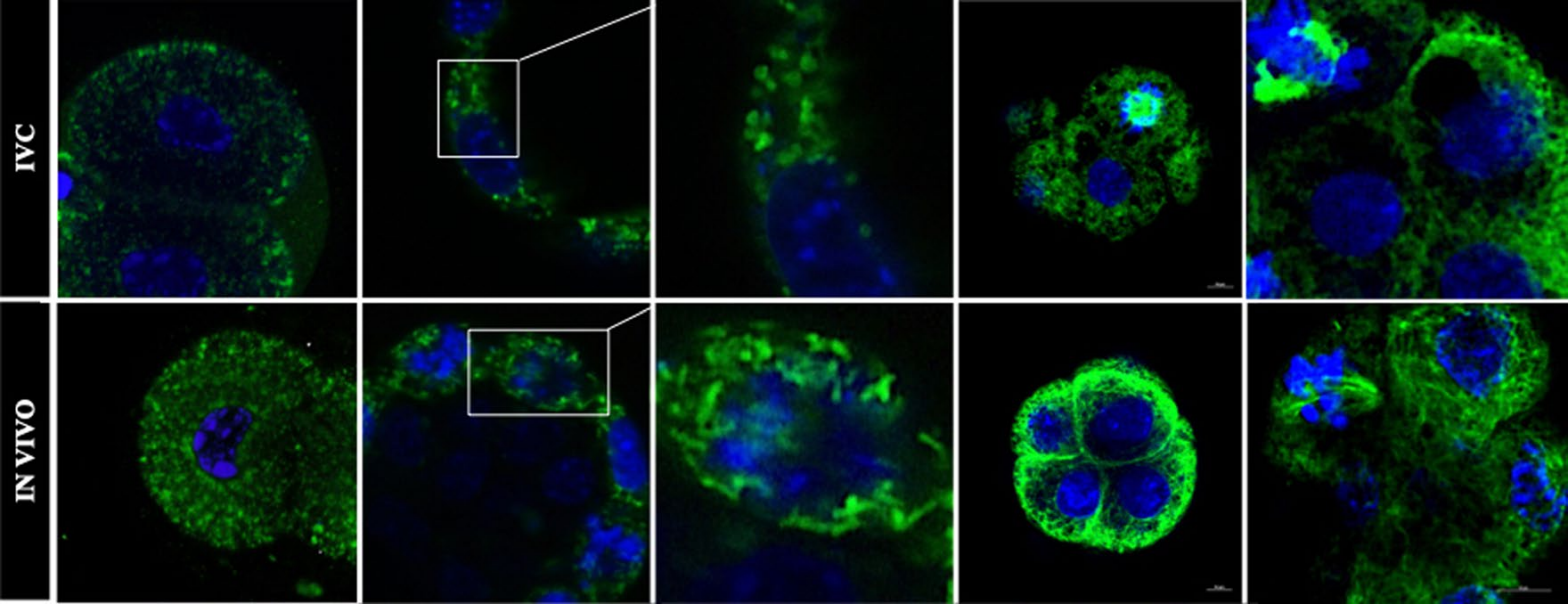
Mitochondrial distribution in mouse IVC (upper line) and IN VIVO developed (bottom line) 2-cell- (left column) and blastocyst (middle and right column) stage embryos. Embryos were stained with Mitotracker Green (green) for mitochondria and with Hoechst (blue) for nuclei visualization. The central column shows an enlarged of the white square from pictures of the middle columns. Scale bar: 10 µm.

Results shown that mitochondria in 2-cell IVC embryos were less numerous compare to IN VIVO, while the localization and distribution do not differ between the groups. Mitochondria were homogenously distributed in both blastomers in both groups (Fig. 2, left column). Major differences were observed at the blastocyst stage (Fig. 2, middle and right column). The proper morphology of mitochondria in cells is tubular shape. Mitochondria in IN VIVO blastocysts were numerous and formed long extended tubular networks across the cells (Fig. 2, bottom pictures). Most of the mitochondria in IVC embryos (Fig. 2, upper pictures) had a spherical or oval shape, and do not create elongated network, we classified them as “fragmented” (Fig. 2, upper pictures, central column). While the mitochondria were visible as shorter and smaller have been considered as fragmented.

**Figure 3:**
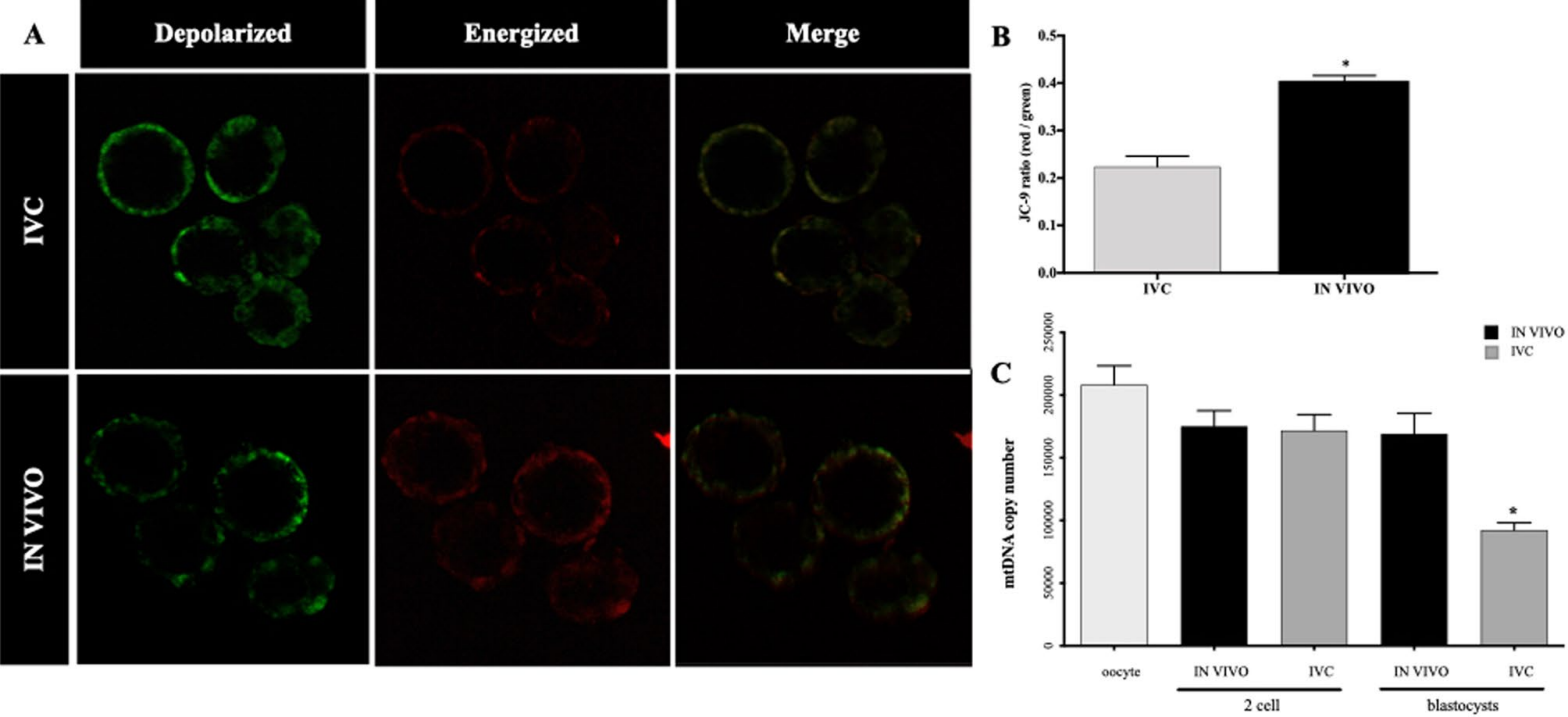
Assessment of mitochondrial depolarization using JC-9 die. (**A**) Representative images of mouse blastocysts stained with JC-9 dye of *IVC* (upper) and IN VIVO (bottom). Monomers of JC-9 were detected as green fluorescence while dye aggregates yielded red to orange colored emission. Scale bars: 10 µm. (**B**) Statistical analysis of the red and green mean fluorescence intensity ratios revealed that mitochondrial membrane potential in IVC embryos was significantly lower than that in the in vivo-developed blastocysts. (**C**) Assessment of mtDNA copy number in in vivo-developed and *IVC* embryos at the 2-cell- and blastocyst stage. The graph presents mtDNA copy number per embryo (n = 10/group); *significant difference at *P* < 0.05.

We next assessed mitochondrial membrane potential in cleavage- (2-cell) and blastocyst stage IVC and IN VIVO developed mouse embryos, using JC-9 staining. We have not observed any differences between the groups in 2-cell stage embryos (data not shown), while strong differences were observed in IVC blastocyst vs IN VIVO groups. Mitochondria with low membrane potential formed aggregates, mainly in the perinuclear regions, while highly polarized mitochondria localize to the periphery of the cell (Fig. 3A). The intensity ratio of red: green hence, mitochondrial membrane potential at the blastocyst stage was significantly lower in IVC embryos when compared to IN VIVO developed blastocysts (Fig. 3B; *P* < 0.05).

Thus, our results indicate that IVC causes mitochondrial dysfunction that might explain the poorer developmental potential of embryos cultured in vitro.

### Analysis of mtDNA copy number in IVC and in vivo-developed embryos, and cell number in blastocyst stage embryos

To determine if in vitro culture affected the quantity of mtDNA present in embryos at the 2-cell and blastocyst stages, assessment of mtDNA copy number was performed (Fig. 3C). We found no difference in mtDNA copy number at the 2-cell stage embryos between IVC (171,845.52 ± 15,191.56, *P* > *0.05*) and those that had developed IN VIVO (175,060.2 ± 12,500.96). However, mtDNA copy number was significantly higher in in vivo-developed blastocysts (169,103.92 ± 16,322.41, *P* > 0.05 when compared to IVC counterparts (92,336.65 ± 5860.04, *P* > 0.05; Fig. 3C).

In terms of mtDNA copy number per cell, levels were slightly lower in IVC blastocysts than in the IN VIVO developed (2949.66 vs. 3166.74, respectively).

This observation shows that IVC affect not only the pattern of mitochondrial distribution and activity, but also mtDNA copy number.

### Effect of IVC on mitochondrial dynamics and motility in mouse embryos

**Figure 4:**
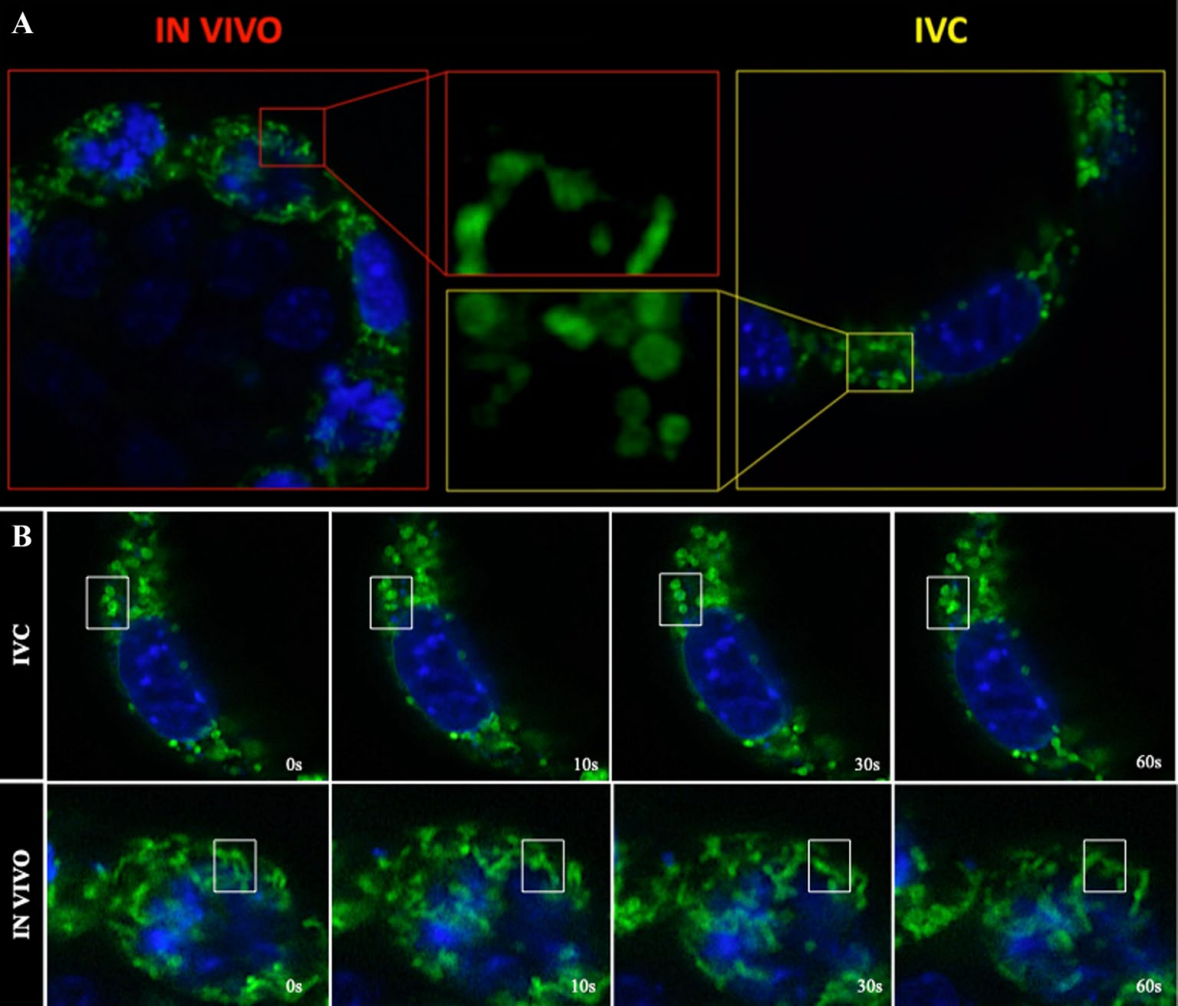
Mitochondrial dynamics and motility. (**A**) Shape of the mitochondria of the IN VIVO (left) and in vitro (right) blastocysts. Central pictures shown an enlarged of the yellow and red squares; (**B**) Images collected during the time lapse registation of IVC and IN VIVO blastocysts. Consucutive frames taken over 20 s show an enlargen section of live cells over time; white frames show examples of localized motion in the INC and IN VIVO blastocyst cell.

Mitochondria are dynamic organelles, undergoing constant migration and morphological changes. Using time-lapse confocal microscopy, we searched for aberrations in mitochondrial dynamics in association with the embryonic development environment. Most mitochondria in embryos developed in vivo were tubular (Fig. 4A), directed radially, and moved back and forth along their long axis on radial tracks (Video 1; Video 3). Several apparent fusion and/or fission events were observed for most mitochondria during 15-min recordings (Video 3; Fig. 4B). However, mitochondria in cells of IVC embryos displayed dramatic alteration in their mobility (Video 2, Video 3). In IVC blastocysts, ovoid or spherical mitochondria (Fig. 4) underwent fusion events less frequently (Video 3, Fig. 4B).

**Figure 5:**
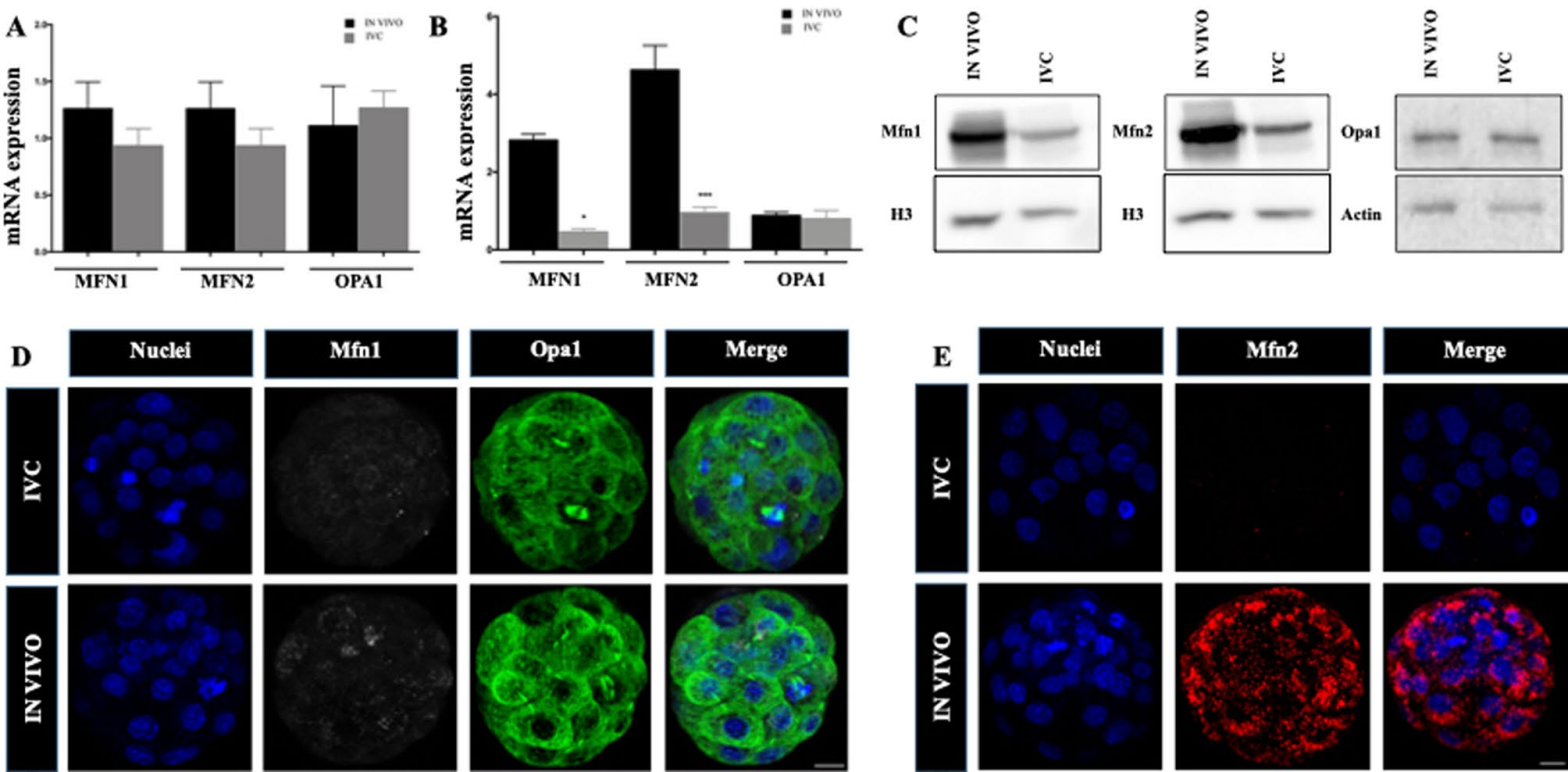
Mitochondrial fusion (Mfn1, Mfn2) and fission (Opa1) mRNA and proteins expression in mouse embryo. (**A**) Mfn1, Mfn2 and Opa1 gene expression detected by qPCR in 2-cell-stage and (**B**) blastocyst stage (**B**) embryos; (**C**) Protein expression of Mfn1, Mfn2 and Opa1 in IVC and IN VIVO blastocysts; (**D**) Mfn1 and Opa1 and (**E**) Mfn2 protein localisation determined by immunostaining in in vivo-developed and IVC blastocysts. Scale bars: 10 µm.

To evaluate whether IVC affects mitochondrial fusion, we first assessed the expression levels of mitochondrial fusion-related mRNA, including *Mfn1*, *Mfn2* and fission *Opa1* in cleavage- (2-cell) and blastocyst stage embryos in both groups. No differences were found in 2-cell embryos (Fig. 5A), whereas *Mfn1* and *Mfn2* mRNA level was statistically lower in IVC blastocyst when compared to the in IN VIVO one while no differences were observed regarding *Opa1* gene (Fig. 5B). Then, protein expression and localisation of mitochondrial fusion (*Mfn2* and *Mfn1*) and fission (*Opa1*) in blastocyst stage IVC and in IN VIVO embryos were then evaluated (Fig. 5C–E). Western blot (Fig. 5C) and immunostaining analysis (Fig. 5D,E) shown lower expression of Mfn1 and Mfn2 in IVC embryos while similar display of Opa1 was observed in both analysed groups. Mfn1 and Mfn2 protein was homogenously distributed in in vivo-developed blastocysts in all the cells, while protein aggregates, mainly peripheral, were noted in cells of in IVC embryos. Opa1 protein shown similar, homogenous distribution in both groups (Fig. 5D).

### Selected mitochondrial genes and proteins involved in mitochondrial physiology and mitophagy

**Figure 6:**
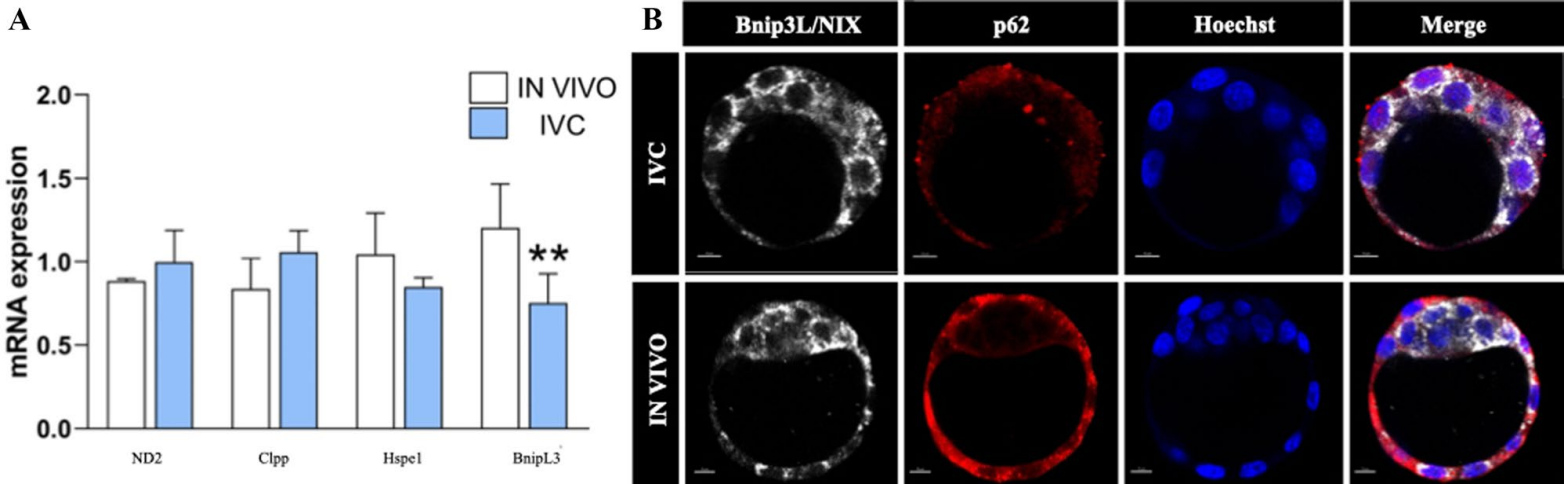
Expression of selected genes and proteins involved in mitochondrial function and mitophagy. (**A**) genes involved in mitochondial protein quality control (Clpp and Hspe1), electron transport chain (mtND2) and mitophagy (BnipL3) **p = 0.0018; (**B**) Protein expression of BnipL3 and p62 in IVC and IN VIVO blastocysts. Scal bars: 10 μm.

The level of selecetd genes knows to play important role in mitochondral physiology (*Clpp, Hspe1* and *mtND2*) and mitophagy (*BnipL3*) were measured. Among the genes tested only *BnipL3* was downregulated (P = 0.0018) while *Hspe1* showed a trend for lower level (P = 0.053) in IVC groups compare to control (Fig. 6A). To evaluate whether in vitro culture affects/induce the mitophagy we assessed protein expression and localisation of mitophagy proteins (BnipL3 and p62) in IVC and IN VIVO blastocysts (Fig. 6B). Immunostaining analysis did not show differences in the expession and localization of BnipL3 (left column) in analysed groups while strong differences were observed in the expression of p62 protein. Results shown very low (close to background) expression of p62 in IVC embryos. In IN VIVO blastocysts p62 was strongly expressed but manily in the trophoblast cells (Fig. 6B; second column from left).

## Discussion

In the present study, we have demonstrated that in vitro culture of mouse embryos significantly affects the functionality of the mitochondria and might be responsible for the impaired embryonic development observed here and by others^7,15,16^

In vitro culture of mammalian embryos is a very important tool when studying the early stages of embryogenesis and the environment in which early embryos can develop and form blastocysts. Moreover, proper embryo culture is one of the main factors ensuring that infertility programs are successful at the human clinics^1,17^*.* A number of reports have shown the influence of culture medium (oxygen concentration, medium components etc.) at the in vitro culture stage on embryonic development, fetal growth, and the postnatal health of the offspring^15,16,18^.

The first observation that we have made was, that number of mitochondria in IVC 2-cell embryo was lower compare to IN VIVO one (Fig. 2). This alteration would very likely affect further embryonic development^19^. It is important to point out here, that the embryo depends on the function of existing mitochondria present at ovulation till blastocyst stage^20^. As cell division begins, the total number of mitochondria within each blastomere decreases due to dilution without new mitochondrial biosynthesis^21^. Consequently, any adverse influence on mitochondrial numbers and function will negatively impact the development of the pre- and post-implantation embryo. An implication of this finding is that as long as the segregation of mitochondria is homogenous between blastomeres, each cleavage from the zygote to the blastocyst halves the number of mitochondria per cell^17^. Moreover, it has been shown that homogeneously distributed mitochondria are found in normal in vivo delivered cleaving embryos, whereas in developmentally arrested embryos they aggregate in the perinuclear/pericortical regions, likely as a result of aberrant cytoskeletal and microtubular organization^4,8,21^.

Importantly, the progression through preimplantation development can be predicted not only by mitochondrial distribution within the cell but also by their number. In fact, we have confirmed that total number of mitochondria with active membrane have decreased in IVC embryos through their development from the 2-cell stage to the blastocyst formation, confirming previous reports in mouse model^23,24^. In human and mouse cleaving embryos, mitochondria with low membrane potential tend to aggregate in the perinuclear region, as was the case with the IVC blastocysts in our study (Fig. 3A). Highly polarized mitochondria, such as in the in IN VIVO blastocysts in our study (Fig. 3A,B), localize in the periphery of the cells, without direct contact with the neighbouring cell^4^. The IVC blastocysts in our work were characterized not only by a lower total number of active mitochondria, but also by a reduced copy number of mtDNA. Each mitochondria has 2–10 copies of mitochondrial DNA. mtDNA copy number is a critical component of overall mitochondrial health and is a measure of mitochondrial DNA (mtDNA) levels per cell, while not a direct measure of mitochondrial function, is associated with mitochondrial enzyme activity and adenosine triphosphate production.

Morphology of the mitochondria, maintained by a balance between their fision and fusion, is critical for proper cellular function. Numerous studies have suggested that an imbalance in mitochondrial fusion/fission might lead to lower blastocysts quality^25,26^. It is known that Mfn2 plays a central role in the fusion process and have been reported to also play an important role in embryonic development^7^. In this work, we showed low gene expression of Mfn1 and Mfn2 and of the protein itself in IVC embryos, which might suggest an unsettled fusion process. Interestingly, we have observed that fission process was not affected in our case. mRNA and well as protein of Opa 1 level was not differing between the groups. Furthermore, the time-lapse videos revealed striking alterations in mitochondrial mobility in the IVC blastocysts cells. Most mitochondria in the in IN VIVO blastocysts cells were tubular, directed radially, and moved back and forth along their long axis on radial tracks.

Since the mitochondrial quality control plays a central role in cellular homeostasis, a couple of factors involved in mitochondrial biogenesis (Clpp, Hspe1 and mtND2) and mitophagy (BNIP3L/Nix and SQSTM1/p62) were analysed. The results showed no statistical differences in Clpp, Hspe1 and mtND2 genes while interesting observations were obtained where mitopgahy proteins were studied. In the IVC blastocyst *BNIP3L/Nix* mRNA level was statistically lower when compared to the IN VIVO blastocysts (Fig. 6A). Then, immunostaining analysis showed no differences in the expression of BNIP3L/Nix. Drastic differences in the expression of p62 was observed in the both analysed groups. Moreover only trophoblastic cells express p62 protein while in the ICM cells very low positive signal was detected (Fig. 6B). Besides mitochondrial biogenesis, mitochondrial elimination via the autophagosome-lysosome pathway (mitophagy), plays a crucial role in maintaining the cellular steady state of mitochondrial functions. It has been shown that SQSTM1 (p62) localization plays a role in regulating mitochondrial morphology, genome integrity and mitochondrial import of a key transcription factor^27^. In vitro environment has a big impact not only on efficiency, number, fusion/fission and homeostasis of mitochondria but also for efficient removal of poor quality mitochondria in preimplantation mouse embryos.

As already has been mentioned before, efficient mitochondrial function and turnover depends on continuous structural remodeling through fusion and fission^28^. Daughter mitochondria produced by fission can either maintain intact membrane potential or become depolarized. Depolarized mitochondria may restore their membrane potential and return to normal fusion/fission equilibrium or remain as non-fusing mitochondria to be eliminated by autophagy. It has been proposed that fission acts as an autophagic checkpoint^29^. Depolarized mitochondria change their morphology to a more fragmented form. Completion of the mitophagy pathway appears to be dependent on p62^30^.

Mouse zygotic genome activation (ZGA) takes place at the 2-cell stage^31^. As can be expected, this process dependents on many structural and epigenetic changes of the maternal and paternal genomes, that are reprogrammed to facilitate development of the embryo^32^. Such major reprogramming of the genome requires metabolites, such as α-ketoglutarate (α-KG) that is essential for protein and DNA demethylation, acetyl-CoA that is required for protein acetylation, ATP that is required for phosphorylation of substrates, and UDP-GlcNAc needed for glycosylation^33^. Production of each of these depends on mitochondrial enzymes that are driving the tri-carboxylic acid (TCA) cycle, and utilization of pyruvate by pyruvate-dehydrogenase. Properly functioning mitochondria are necessary from the very beginning of the existence a new organism as an energy source of extremely dynamically developing organism (polarization, compaction, cavitation, differentiation as well as implantation).

Studies on mitochondrial metabolism have identified an intriguing link between energy demand/supply balance and mitochondrial architecture, suggesting that inappropriate culture conditions might inhibit mitochondrial functions, with negative effects on embryonic development. In our work, we have confirmed this hypothesis. We have shown, to our knowledge for the first time, that a culture medium commonly used for in vitro mouse embryo culture negatively affects mitochondrial functionality and motility. These abnormalities strongly influence embryonic development and quality. Our observations suggest that studies aimed at improving the quality of IVC media and culture conditions should consider the inclusion of metabolites/factors responsible for mitochondrial activity.

## Materials and methods

Unless otherwise stated, all inorganic and organic materials were purchased from Sigma.

### Animals and ethics statements

The experiments were performed according to the Polish Governmental Act for Animal Care and were approved (WAW2/62/2017) by the Local Ethics Committee for Animal Care at Warsaw University of Life Sciences and the reporting in the manuscript follows the recommendations in the ARRIVE guidelines.

All experiments were carried out on F1 (C57Bl10 x CBA/H) mice in the Institute of Genetics and Animal Biotechnology of the Polish Academy of Sciences. Animals were housed in 30.5 × 13 × 11 cm cages, kept in a temperature-controlled room with a 12 h/12 h light/dark cycle (light between 06.00 and 18.00). Food (Labofeed H, Kcynia, Poland; metabolic energy of 13.0 MJ/kg) and water were available ad libitum.

### Embryos

For embryo collection, donor F1 females (3–5 months old) were stimulated for ovulation by intraperitoneal injection of 7.5 IU (0.1 mL) PMSG (Pregnant Mare Serum Gonadotropin; Folligon, Intervet, Boxmeer, The Netherlands), followed 48 h later by 7.5 IU (0.1 mL) of hCG (human Chorionic Gonadotropin; Chorulon, Intervet). Mice were then naturally matted. One part of the animals were scarified by cervical dislocation 24 h after hCG injection, and zygotes stage embryos were collected. Recovered embryos were washed three times in M2 medium and cultured in vitro in standard KSOM medium (KCl-enriched simplex optimized medium; Specialty Media, Phillipsburg, NJ, USA), supplemented with amino acids and 4 mg/mL BSA, under paraffin oil in an atmosphere of 5% CO_2_ in air at 37.5 °C. Incubation was done till the 2-cell and/or blastocyst stages (IVC). As a control, embryos at 2-cell stage (48 h post hCG injection) and blastocysts (92 h post hCG injection) were flushed and directly used for analysis (IN VIVO).

### Assessment of cell number of IVC and IN VIVO embryos

IVC and IN VIVO embryos at the blastocyst stage (ten embryos per group) were fixed with 4% paraformaldehyde (PFA), permeabilized in 0.1% Triton X-100, and then stained with 5 µg/mL of Hoechst 33,342. Stained blastocysts were visualized using a confocal microscopy (Nikon Eclipse Ti-E), using NIS-Elements Confocal software (Nikon). Images were analyzed using IMARIS 6.0.1 software (Bitplane AG, UK). Nuclei were identified using the ‘spot’ option in the software, with an estimated diameter of 7–10 μm in the Hoechst channel. The number of nuclei identified by the software was adjusted manually.

### Mitochondrial staining

Mitochondrial distribution and segregation were quantified using Mitotracker Green staining. 2-cell and blastocysts were incubated with 100 µM of Mitotracker Green FM (Invitrogen, Molecular Probes, Milan, Italy) in M2 medium for 30 min at 37.5 °C. Then, embryos were washed twice with PBS, and then counterstained with 5 µg/mL of Hoechst 33342 for 10 min. Mitochondria distribution in embryo cytoplasm appeared as small individual fluorescent dots or as aggregates. All samples were examined by confocal microscopy (Nikon A1R), using NIS-Elements Confocal software (Nikon). Images were analyzed using IMARIS 6.0.1 software (Bitplane AG, UK).

### Immunofluorescence staining

The zona pellucida was removed using acidified Tyrode’s solution. Embryos were fixed in 4% paraformaldehyde in phosphate-buffered saline (PBS) with 0.1% Tween 20 and 0.01% Triton X-100 for 10 min at room temperature (RT), permeabilised in 0.55% Triton X-100 in PBS for 15 min at RT, washed 4 times (5 min each) in PBX (PBS with 0.1% Triton X-100) and then blocked in 20% fetal bovine serum in PBS for 1 h at RT. The primary antibodies were used: anti-Mfn2 mouse monoclonal antibody (ab56889, Abcam); anti-Mfn1 mouse monoclonal antibody (ab126575, Abcam); anti- OPA1 rabbit monoclonal (ab157457) and anti-Sox2 rabbit polyclonal (ab97959, Abcam); anti-BnipL3 (ab109414, Abcam); anti-SQSTM1/p62 (sc-28359, Santa Cruz) at dilutions of 1:100 in 20% fetal bovine serum in PBS. Embryos were incubated in primary antibodies solution at 4°C overnight. After that embryos were washed 4 times (5 min each) in PBX and then blocked in 20% fetal bovine serum in PBS for 1 h at RT. The secondary Alexa Fluor-conjugated antibodies (Invitrogen) were used at a dilution of 1:500 in 20% fetal bovine serum in PBS. Embryos were incubated at 4°C for 75 min, then they were washed 4 times (5 min each) in PBX. DNA was visualised using Hoechst 33342 (5 μg/mL; Molecular Probes).

### Quantitative real-time polymerase chain reaction

Isolation of total RNA was performed using High Pure miRNA Isolation Kit (Roche Applied Science, Germany) according to manufacturer's protocol. 10 embryos from a given developmental stage were used for RNA isolation. The embryos were previously frozen in 10 µl of nuclease-free water in liquid nitrogen and placed at − 80 °C. Synthesis of cDNA was performed using Transcriptor High Fidelity cDNA Synthesis Kit (Roche Applied Science, Germany) according to manufacturer's protocol. For the reaction, we used 100 ng of RNA. qRT-PCR reaction was performed using Light Cycler FastStart DNA Master SYBR Green I qRT-PCR kit (Roche Applied Science, Germany), according to manufacturer's protocol, on Roche 96 thermocycler. Each reaction was followed by a melting curve analysis to ensure the specificity of amplification. The efficiency of the reactions was analysed. Primer pairs were designed using the Primer-BLAST tool (NCBI, USA; www.ncbi.nlm.nih.gov/tools/primer-blast/) using Ref Seq IV.80 database that contains a complete list of known mouse transcripts. Although the primers did not yield non-specific products as revealed by the BLAST analysis, we performed standard gel electrophoresis and melting curve analysis. We used *H2afz* and *18S* as a reference genes. Detailed information about primers sets are presented in Supplementary Table 1 (Supplement_Table_1).

### mtDNA copy number analysis

Total DNA was extracted from embryos at the 2-cell and blastocyst stage (4 embryos per group), using the QIAamp DNA Micro Kit (Qiagen, Hilden, Germany), according to the manufacturer’s instructions. Primers were designed for the genomic NADH dehydrogenase subunit 2 (ND2) region (F: ATCCTCCTGGCCATCGTACT; R: ATCAGAAGTGGAATGGGGCG, Tm 60 °C, product size 136). Quantification of mtDNA copy number was performed by qPCR, using Roche 96 thermocycler with Light Cycler FastStart DNA Master SYBR Green I qPCR kit (Roche Applied Science) using the standard curve method.

The ND2 region was amplified from total DNA and visualized on 1.5% agarose gel. The PCR products were purified using the QIAquick Gel Extraction Kit (Qiagen, Hilden, Germany), according to the manufacturer’s instructions to generate standards for the qPCR. Based on the DNA concentration measured with a Nanodrop c2000 system (Thermo Scientific, USA), serial tenfold dilutions of a DNA with a known concentration (standards) were generated. Each standard was used as a separate template for a qPCR reaction to produce the appropriate standard curve with the Light Cycler 96 software (Roche, Switzerland). The efficiency of the reaction was analyzed. Finally, each PCR run with experimental samples contained one DNA standard of a known concentration. For each run, a standard curve was generated from tenfold serial dilutions (10^−1^ to 10^−8^). Each sample was analysed in triplicate to obtain the mean number of mtDNA copies.

### Western blot

Mitochondria were extracted from the IN VIVO and IVC blastocysts using Mitochondrial Isolation Kit (Abcam, ab110168). The equal amount of protein samples was loaded and SDS-PAGE (10% sodium dodecyl sulfate polyacrylamide gel electrophoresis) was performed. After that, the proteins were transferred to a PVDF membrane (Merck-Millipore, USA) and blocked with Tris buffered saline containing 0.05% Tween-20 (PBST) and 5% non-fat dry milk for 1 h at RT. After blocking, the membranes were incubated with corresponding antibodies [Mfn1, Mfn2, Opa1 and β-actin (sc. 1615, Santa Cruz) or H3 (ab 1791, Abcam) which were used as a loading controls)] at 1:1000 at 4 °C overnight, washed in PBST, followed by incubation with the corresponding horseradish peroxidase-conjugated secondary antibodies for 1 h at RT. Visualization of the proteins was detected with ECL chemiluminescence (IN VIVO and IVC blastocysts: *n* = 100 for per lane).

### Mitochondrial membrane activity

JC9 is a cationic dye which binds to mitochondria and emits green fluorescence (∼525 nm) independent of mitochondrial membrane potential (MMP). In the case of mitochondrial membrane hyperpolarization, JC9 aggregates and gives off red fluorescence (∼590 nm). Therefore, the intensity ratio of red: green can be used to measure the MMP and indicates the mitochondrial activity^25^.

IN VIVO and IVC embryos were loaded with M2 medium containing JC-9 dye (final concentration: 2 μg/ml) and incubated at 37 °C for 25 min. Then, embryos were washed twice with PBS, counterstained with 5 μg/mL Hoechst 33,342, and observed under confocal microscopy (Nikon Eclipse Ti-E), using NIS-Elements Confocal software (Nikon). After incubation blastocysts were live- imaged under a 63X objective lens with a zoom factor of 1.6X. Both green and red channel images were captured. ImageJ software was used to measure the intensity of green and red fluorescent signals and the ratio of red/green fluorescent intensity was calculated.

### Statistical analysis

Data were analysed using GraphPad Prism (Version 6.01, GraphPad Software, Inc, CA, USA). Statistical analyses of total cell number, mtDNA copy number, and mRNA expression rate were based on three replicates per experiment and were compared by one-way ANOVA. Differences with *P* < 0.05 were considered statistically significant.

## Supplementary Information

Supplementary Information 1.

Supplementary Information 2.

Supplementary Video 1.

Supplementary Video 2.

Supplementary Video 3.

Supplementary Information 3.

## Data Availability

All data supporting the results reported in the article can be found as supplementary files.
