# Author Correction: Mitochondrial function and intracellular distribution is severely affected in in vitro cultured mouse embryos

**DOI:** 10.1038/s41598-022-25820-z

**Published:** 2022-12-08

**Authors:** Marta Czernik, Dawid Winiarczyk, Silvestre Sampino, Paweł Gręda, Salvatore Parillo, Jacek Andrzej Modliński, Pasqualino Loi

**Affiliations:** 1 Institute of Genetics and Animal Biotechnology, Polish Academy of Sciences, ul. Postepu 36A, Jastrzębiec, Poland; 2 Faculty of Veterinary Medicine, University of Teramo, Via Balzarini 1, Teramo, Italy; 3 Department of Morphological Sciences, Faculty of Veterinary Medicine, Warsaw University of Life Sciences, Warsaw, Poland

Correction to: *Scientific Reports* 10.1038/s41598-022-20374-6, published online 27 September 2022

The original version of this Article contained spelling errors in the names of the authors: Marta Czernik, Dawid Winiarczyk, Silvestre Sampino, Paweł Gręda, Salvatore Parillo and Pasqualino Loi, which were incorrectly given as: Czernik Marta, Winiarczyk Dawid, Sampino Silvestre, Greda Pawel, Parillo Salvatore and Loi Pasqualino.

The original Article has been corrected.

