# Supplementary figures and images for: Mitochondrial function and intracellular distribution is severely affected in in vitro cultured mouse embryos
Source: Sci Rep. 2022 Sep 27;12:16152. doi: 10.1038/s41598-022-20374-6 (PMC9515144; doi:10.1038/s41598-022-20374-6)

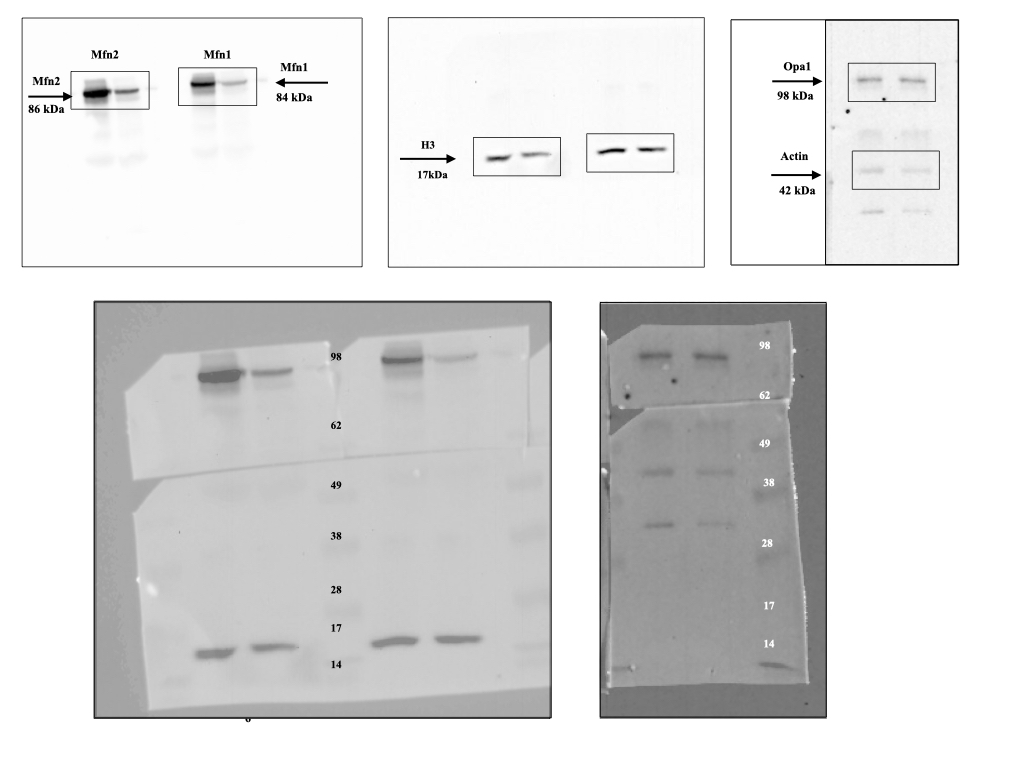

Supplement: Supplementary file 1 — Supplementary Information 1. [file 41598_2022_20374_MOESM1_ESM.jpeg]
