# Supplementary material for: Mitochondrial function and intracellular distribution is severely affected in in vitro cultured mouse embryos
Source: Sci Rep. 2022 Sep 27;12:16152. doi: 10.1038/s41598-022-20374-6 (PMC9515144; doi:10.1038/s41598-022-20374-6)
Supplement: Supplementary file 2 — Supplementary Information 2. [file 41598_2022_20374_MOESM2_ESM.docx]

**Supplement Table 1:** List of the primers used for the gene expression analysis

| Gene | NCBI Reference Sequence | Sequence  (5’->3’) | Annealing Temperature (°C) | Product Size (bp) |
| --- | --- | --- | --- | --- |
| H2afz | NM_016750.3 | CGCAGAGGTACTTGAGTT  TCTTCATCTCCACGTATAGC | 60 | 99 |
| 18S | BK000964.3 | CGCGGTTCTATTTTGTTGGT  AGTCGGCATCGTTTATGGTC | 60 | 119 |
| Mfn1 | NM_024200.4 | GCAGCAGAGAAGAGGGTTTAT  CGTGACCTCCTTGATCTTCTTC | 60 | 123 |
| Mfn2 | NM_001285920.1 | TCAACACCATCAGGGGCCAA  CAGAGGAGAAGTTTCTAGCTGGT | 60 | 136 |
| Opa1 | NM_001199177.1 | ACTTCTTCACTGCAGGTCCCAA  GTCTGACACCTTCCTGTAATGCT | 60 | 146 |
| Oct4 | M_013633.3 | GGTGGAACCAACTCCCGAGG  ACCTTTCCAAAGAGAACGCCC | 60 | 150 |
| Sox2 | AB108673.1 | TTTGTCCGAGACCGAGAAGC CTCCGGGAAGCGTGTACTTA | 60 | 146 |
| Nanog | AY278951.1 | TGATTCAGAAGGGCTCAGCA  GCCCCACATGGAAAGGCTTC | 60 | 115 |
| Hspe1 | NM_008303.4 | GGTGGCATTATGCTTCCAGA  ACAGGCTCAATCTCTCCACT | 55 | 126 |
| ND2 | NM_133667.2 | ACCCATCCCTCACTCTACTCA  GTGGAAGGCCTCCTAGGG | 55 | 143 |
| Clpp | NM_017393.2 | ATCTACGCCAAACACACCAA  GACCTTGTCCAAGATGCCAA | 55 | 132 |
| BnipL3 | NM_009760.4 | AACAGCACTCTGTCTGAGGAA  TGTCAGACGCCTTCCAATGT | 55 | 121 |
