# Supplementary material for: Mitochondrial function and intracellular distribution is severely affected in in vitro cultured mouse embryos
Source: Sci Rep. 2022 Sep 27;12:16152. doi: 10.1038/s41598-022-20374-6 (PMC9515144; doi:10.1038/s41598-022-20374-6)
Supplement: Supplementary file 6 — Supplementary Information 3. [file 41598_2022_20374_MOESM6_ESM.docx]

**VIDEO 1. Mitochondrial dynamics in IN VIVO blastocysts**

**VIDEO 2. Mitochondrial dynamics in IVC blastocysts**

**VEDEO 3. Mitochondrial dynamics IVC vs IN_VIVO**
